# A commentary of “Initial DNA melting in human DNA replication initiation”: Top 10 Scientific Advances of 2023, China

**DOI:** 10.1016/j.fmre.2024.03.007

**Published:** 2024-03-22

**Authors:** Ningning Li, Ning Gao

**Affiliations:** State Key Laboratory of Membrane Biology, Peking-Tsinghua Center for Life Sciences, School of Life Sciences, Peking University, Beijing 100871, China

DNA replication is a complicated process involving several dozens of factors. The molecular machine of eukaryotic DNA replication, replisome, consists of at least three essential modules: one replicative DNA helicase CMG (Cdc45-MCM-GINS) and two sets of DNA polymerase complexes responsible for the synthesis of the leading and lagging strands, respectively. As the core of CMG, MCM is a ring-shaped helicase motor composed of six homologous AAA+ ATPase subunits, Mcm2 to Mcm7. The human genome is enormous, with over 3 billion base pairs. To efficiently duplicate the entire genome within a limited time, DNA replication is initiated from more than 25,000 discrete sites simultaneously. Therefore, DNA replication initiation is tightly regulated to ensure that the duplication of the genome occurs only once per cell division cycle. Abnormalities in DNA replication lead to genomic instability, which is a hallmark of many types of cancer. Oncogene activation could also interfere with replication initiation, which triggers replication stress and drives genomic instability and tumorigenesis [1]. In turn, many DNA replication initiation factors are aberrantly expressed in cancer cells, presenting promising targets for anti-cancer drug development, and potential biomarkers for early cancer diagnosis [1]. For example, MCM subunits have been developed as biomarkers for the early detection of many common cancers [2]. In addition, Dbf4-Dependent Kinase (DDK), a critical kinase in replication initiation regulation, is emerging as a target for anti-tumor chemotherapy. Small-molecule DDK inhibitors have been developed, including TAK-931 (Takeda Pharmaceutical Company) that is already in clinical trials. On the other hand, defects in replication initiation can cause congenital human diseases [2]. The inherited disorder Meier-Gorlin syndrome (MGS) is linked to mutations in the replication initiation factors *ORC1, ORC4, ORC6, CDT1 and CDC6*. A missense variant in *MCM2* has been implicated in familial deafness. Therefore, DNA replication initiation has remained a research focus in fundamental biology for decades.

DNA replication initiation is cell cycle dependent. In the G1 phase, the origin recognition complex (ORC) recognizes the initiation site and recruits two copies of the ring-shaped Mcm2–7 hexamer to assemble an inactive MCM double hexamer (MCM-DH) in a head-to-head configuration encircling the double-stranded origin DNA (dsDNA) [3]. Helicase activation occurs in the G1/S phase and is tightly controlled by two kinases, DDK and cyclin-dependent kinase (CDK). Phosphorylation of initiation factors by these two kinases leads to the formation of two active CMG helicases. Thus, two sets of replisomes are assembled with CMG as the core and they replicate DNA bi-directionally from the replication origin [3]. During the elongation phase of DNA replication, MCM encircles and translates along the leading strand of DNA to unwind the dsDNA, generating two ssDNA templates for DNA synthesis. However, the DNA within the central channel of the assembled MCM-DH in the initiation phase is still in double-stranded form. How and when the dsDNA is melted and how the unwound lagging strand DNA is extruded outside the chamber are largely unknown. These processes are critical for regulation of replication initiation and replisome assembly. Previous structural studies on endogenous yeast MCM-DH (yMCM-DH) and *in vitro* reconstituted dsDNA-containing yMCM-DH [4,5] have shown that two MCM hexamers form a narrow and kinked central channel around the hexamer interface, but the DNA within the channel remains in the double-stranded form. Despite extensive efforts spanning over four decades, these questions remain unanswered.

Most of our knowledge about the mechanism of eukaryotic DNA replication comes from the studies in the budding yeast model system. However, the mechanism of replication initiation in metazoan species, including humans, is considerably different from that in yeast. One well-known example is the mechanistic difference in the origin DNA recognition by ORCs from different species, despite their conservation in sequence and structure [3]. Although the origin DNA in budding yeast contains a highly conserved consensus sequence, such conservation is not found in metazoan species. In humans, replication origin labeling is more dependent on specific nucleosome modifications and higher-order chromatin features. Additionally, the regulation of human ORC activity is more intricate than that in yeast [6]. To avoid the collision between DNA replication and transcription, DNA replication tends to initiate in non-transcribed regions [7,8]. Recent studies indicate that yeast and humans may employ different strategies to achieve this goal. In yeast, the helicase MCM appears to be directly loaded onto the intergenic regions by ORC [8], whereas the mammalian MCM can be redistributed to the non-transcribed regions by RNA polymerase II [7]. In short, a full understanding of DNA replication initiation in human cells is not only of significance in biology but also has great therapeutic potential.

A team led by Dr. Yuanliang Zhai made a significant breakthrough by providing a structural insight into the initial melting of origin DNA in human cells [9]. They determined a 2.6-Å cryo-EM structure of endogenous human MCM-DH (hMCM-DH) purified from chromatins and found that a stretch of dsDNA is stably bound in the central channel of DH (Fig. 1). The overall kinked configuration of the central channel of hMCM-DH is similar to that of yMCM-DH, but the human one exhibits a notably narrower channel around the hexamer interface, measuring 13 Å in diameter. More importantly, the origin DNA was partially melted at the hexamer interface, forming an initial open structure (IOS) on dsDNA, in which one base pair was completely separated. This structure likely captured the very early moment of origin melting and indicates that the initial melting in human cells occurs much earlier than helicase activation. This feature of hMCM-DH is in contrast with yMCM-DH and highlights a potential human-specific feature that might be tailored for anti-tumor drug development. Indeed, by mutating the key MCM residues involved in IOS formation, the authors proved that IOS is critical for the assembly of hMCM-DH and replication initiation in cell. This study represents a significant leap towards resolving the long-standing question of origin DNA melting in DNA replication. That is why this research was selected as one of the “Top 10 Scientific Advances of 2023, China”.

**Fig. 1:**
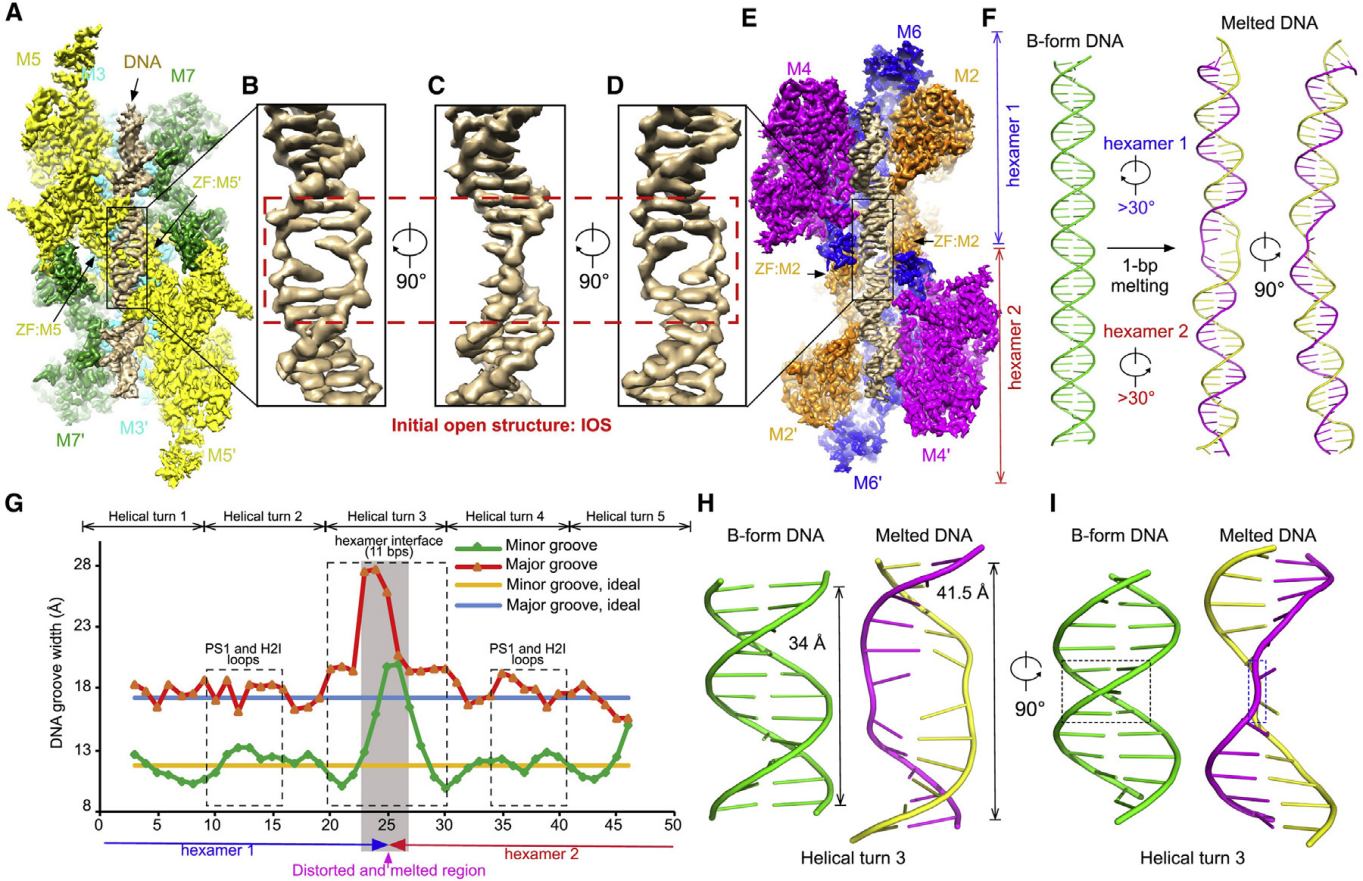
**Conformation of the hMCM-DH-origin DNA open complex**[9].

In short, the study led by Dr. Zhai captured the first snapshot of the initial melting process during replication initiation. However, the mechanisms underlying the expansion of IOS into a replication bubble and the extrusion of the lagging strand from the central channel remain unclear, which merit future investigations. Numerous Chinese researchers have played a pivotal role in addressing fundamental questions in DNA replication, such as investigations into the recognition of replication origin DNA [10], the assembly and activation of MCM-DH [[4], [11]], and the DNA replication-coupled nucleosome assembly [[12], [13], [14], [15]]. A notable recent achievement is the structural study of DNA replication-coupled parental histone recycling [15]. With the rapid evolution of cryo-EM and DNA sequencing-based techniques in recent and upcoming years, it is anticipated that many puzzles in the DNA replication field will be resolved in near future.

## Declaration of competing interest

The authors declare that they have no conflicts of interest in this work.
